# *In utero* elemental tags in vertebrae of the scalloped hammerhead shark *Sphyrna lewini* reveal migration patterns of pregnant females

**DOI:** 10.1038/s41598-020-58735-8

**Published:** 2020-02-04

**Authors:** Claire Coiraton, Felipe Amezcua

**Affiliations:** 1 Posgrado en Ciencias del Mar y Limnología, Universidad Nacional Autónoma de México; Av. Ciudad Universitaria 3000, Coyoacán, Mexico City, 04510 Mexico; 2 Instituto de Ciencias del Mar y Limnología, Universidad Nacional Autónoma de México. Av. Joel Montes Camarena s/n, Mazatlán 82040, Sinaloa, Mexico

**Keywords:** Chemical ecology, Animal migration

## Abstract

Vertebral microchemistry recently allowed to infer the migration patterns of the scalloped hammerhead shark *Sphyrna lewini* in the Mexican Pacific, however conclusions regarding the movements of reproductive females were hindered by the small sample size. Considering that *S. lewini* is a placental viviparous species, maternal supply of nutrients to the embryos might influence their vertebral microchemistry while *in utero* and provide intrinsic markers of the pregnant female environmental histories. This hypothesis was tested before attempting to infer the migration patterns of pregnant females through the analyses of the *in utero* elemental profiles quantified in the vertebrae of coastal young-of-the-year (‘YOY’). Vertebrae were obtained from sharks captured along the Mexican Pacific coast in 2016. Vertebral microchemistry was quantified using laser ablation inductively-coupled plasma mass spectrometry. Elemental signatures at vertebral edge were consistent between each pregnant female and her embryos demonstrating the viability of employing *in utero* elemental signatures as a maternal tag of the gestation-related environmental histories. Analyses of the YOY *in utero* Sr:Ba and Pb:Ca profiles suggested that pregnant females either (1) progressively migrated offshore before quickly returning to coastal habitats before term or (2) remained nearshore during complete gestation. Considering the endangered status of *S. lewini*, current management measures may be insufficient for the sustainable management of the population as pregnant females may be particularly susceptible to fisheries when remaining nearshore or entering coastal habitats prior to pupping.

## Introduction

The scalloped hammerhead shark, *Sphyrna lewini* (Griffith and Smith 1834), is a circumtropical migratory species that uses both oceanic and coastal habitats for its dispersal and reproductive strategy^1^. In the Mexican Pacific, neonate and juvenile stages of *S. lewini* are typically found in shallow estuarine and coastal habitats^2–4^ for their first 3 to 7 years of life^5–7^ before migrating offshore to maximize their foraging opportunities and grow more rapidly to reproductive size^5^. Adults, on the other hand, seasonally form schools near seamounts and oceanic islands^8–10^ and females later return to coastal habitats for parturition (possibly annually^11,12^). Although males do not have such requirement, recent studies indicated that some individuals may also later return to coastal habitats or even remain nearshore for their entire life^7,13^, thus being able to mate opportunistically with females entering coastal waters to give birth^13–15^. Though these findings provided more insights into the life history of males, the present state of knowledge on the migratory patterns of females remains fragmentary, particularly during the gestation.

Microchemical signatures derived from elements deposited in the vertebrae of sharks during growth can reflect changes in the surrounding water chemistry^16–19^, environmental conditions^16,18,20^ and diet^21,22^, and thus serve as discrete site-specific markers^19,23–27^ or time-resolved records of the individual environmental histories when related to growth bands^7,17,19,20,28^. More specifically, it was recently showed that vertebral microchemistry of the scalloped hammerhead shark *S. lewini* could accurately distinguish among individuals from different locations of the Mexican Pacific, whether these had been occupying coastal or more oceanic habitats, and address important questions concerning the recent habitat use and natal origin^7,23^ of the species in the region. Concentrations of strontium ^88^Sr, barium ^137^Ba and lead ^208^Pb were assessed along vertebral transects encompassing complete life histories, which allowed to document several aspects on the migratory patterns of *S. lewini* because these elements were found to reflect the nearshore-offshore movements of the sharks across the apparent salinity gradient^7,17,19,29^ and periods of nearshore residency into contaminated habitats^7,30–32^. Robust conclusions regarding the migration patterns of females, and particularly mature individuals, were however hindered by the small sample size and the lack of discernible pattern in the age-related elemental profiles for some individuals^7^.

As a placental viviparous species, *S. lewini* exhibits high degree of maternal investment in its offspring. During their first weeks of development, embryos derive their nourishment from their yolk-sacs after which these transform to become a highly vascularized placenta that directly provides nutrients to the embryos through the mother’s blood stream^33^. Given the long gestation period of *S. lewini* (*i.e*. 10–11 months^11,12^), maternal supply of nutrients to embryos has the potential to influence the vertebral microchemistry of embryos while *in utero*^7^, at least after placental formation^34^. Accordingly, validating the relationships between maternal vertebral microchemistry and elemental composition of embryos may provide novel proxies for inferring the migration patterns of pregnant females and facilitate further investigations on the species population connectivity through the examination of *in utero* elemental signatures or transect profiles quantified in the vertebrae of coastal juvenile stages^7^, that are a more frequent component of the small-scale fisheries operating along the Mexican Pacific coast when compared to pregnant females^2,3,35^.

The objective of this study was to establish the viability of employing the *in utero* elemental signatures deposited in vertebrae of *S. lewini* as intrinsic markers of the environmental histories of pregnant females during their gestation in the Mexican Pacific. More specifically, this study sought to test the hypotheses that: (1) elemental signatures in vertebrae and (2) elemental profiles encompassing complete *in utero* development were consistent among embryos within each pregnant female’s litter and (3) vertebral microchemistry of embryos reflected that of pregnant females during the gestation in order to (4) attempt to assess the gestation-related movements of pregnant females using *in utero* maternal tags in the vertebrae of coastal juveniles. Only young-of-the-year specimens (age 0) were considered to avoid potential site-specific temporal variations in the water chemistry that might result in different elemental signatures for individuals of different ages originating from the same nurseries^23^.

## Material and Methods

### Sample collection

**Figure 1:**
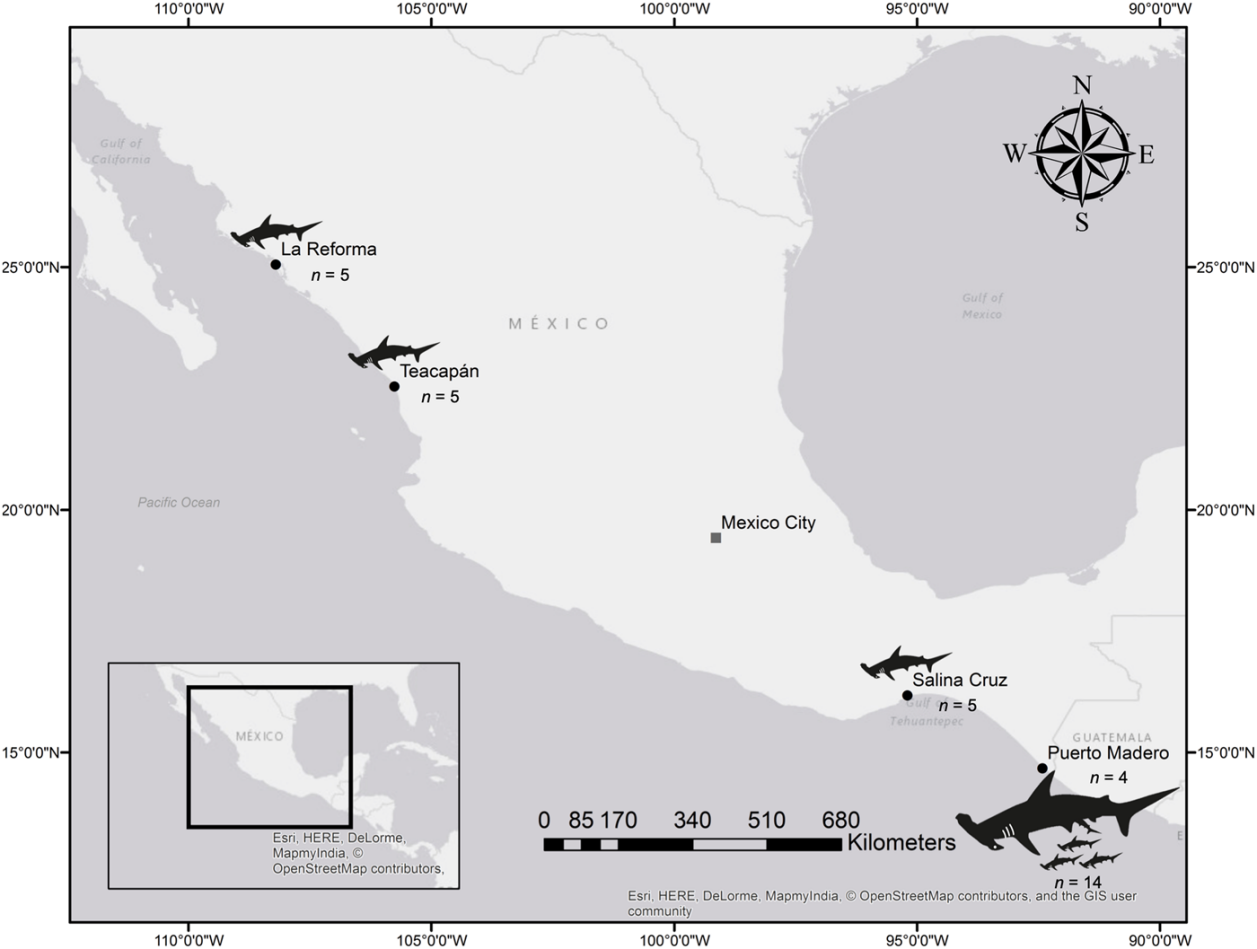
Map of the region of study showing the sampling sites of pregnant females (*n* = 4), embryos (*n* = 14) and young-of-the-year (*n* = 15) specimens of *Sphyrna lewini* in the Mexican Pacific (hammerhead illustrations used with permission ©*Flora pixelia*).

Vertebrae samples from pregnant females of *S. lewini* and their embryos were obtained from specimens captured in April 2016 by the artisanal shark fishery operating off Puerto Madero (Fig. 1; Table 1). Vertebrae samples from young-of-the-year specimens were obtained between August and October 2016 from three artisanal fishery landings along the Mexican Pacific coast, in La Reforma, Teacapán and Salina Cruz (Fig. 1; Table 2). These sampling locations were selected to address the questions of this study because they were reported as important nursery areas for *S. lewini* in the Mexican Pacific^2–4,11,36,37^. Fishers used surface and bottom gillnets and surface longlines. All sample collection was opportunistic and carried out in accordance with relevant national guidelines and regulations. Sex and total length (*L*_T_) were recorded for each specimen and a set of post-cephalic vertebrae was removed and stored frozen until preparation for analyses.

**Table 1:** Codes, total length (*L*_T_; cm) range and mean (±standard deviation) of the 4 pregnant females of *Sphyrna lewini* and their near-term embryos (*n* = 14) captured off Puerto Madero in April 2016.

Female’s litter code	*L*_T females_	*n*_embryos_	*L*_T embryos_
1	291	4	41–43 (42.1 ± 0.9 cm)
2	277	3	41–47 (44.5 ± 3.1 cm)
3	253	3	47
4	240	4	43–49 (46.6 ± 2.6 cm)

**Table 2:** Sampling details, total length (*L*_T_; cm) range and mean (±standard deviation) of the 15 young-of-the-year specimens of *Sphyrna lewini* collected in 2016 in three coastal nurseries of the Mexican Pacific.

Sampling site	Date of capture	*n*	*L*_T_
La Reforma	Sept 2016	5	55–63. (59.4 ± 3.4 cm)
Teacapán	Aug 2016	5	52.8–56.8 (54.2 ± 1.7 cm)
Salina Cruz	Oct 2016	5	49.2–59.6 (55.3 ± 4.5 cm)

### Vertebrae preparation for LA-ICP-MS analyses

Vertebrae were defrosted and the neural arch and extraneous tissue were removed. Individual centra were exposed, thoroughly rinsed, air dried, mounted on wooden holders with polyester resin and later cut into 0.4 mm thick sagittal sections with a Buehler low-speed Isomet saw. Vertebral sections were hand-polished with a series of progressively finer grades of lapping paper (220, 800, and 3 µm grit), ultrasonically cleaned for 5 minutes in Milli-Q water to remove surface contaminants, triple rinsed, dried for 24 hours and randomly affixed with double-sided tape to acid-washed petrographic slides (subsequently referred to as ‘master slides’). One vertebra section from each specimen was used for analyses as it was shown that elemental signatures of *S. lewini* did not differ in individual sharks^23,38^. All cleaning and drying procedures were performed under a Class-100 laminar flow clean hood using trace-metal grade reagents, non-metallic instruments and HNO_3_ acid-washed glass slides to minimize contamination.

### LA-ICP-MS analyses

The elemental composition of the vertebrae of *S. lewini* was quantified using a Photon-Machines Analyte 193 excimer UV laser ablation system (LA), connected to an Agilent Technologies 7500CX quadrupole inductively coupled plasma–mass spectrometer (ICP-MS). Raw data of the ion counts per second (cps) were collected for the following 21 elements: ^7^Li, ^24^Mg, ^43^Ca, ^45^Sc, ^51^V, ^53^Cr, ^55^Mn, ^57^Fe, ^59^Co, ^63^Cu, ^72^Ge, ^85^Rb, ^88^Sr, ^89^Y, ^114^Cd, ^118^Sn, ^137^Ba, ^197^Au, ^208^Pb, ^232^Th and ^238^U with ^43^Ca being quantified for use as internal standard. These were screened in the vertebrae of *S. lewini* because this combination of masses minimizes potential interferences that can arise from isobaric spectral overlap, sample matrix effects, and the presence of molecular ions^39^ and was successfully used in previous studies for inferring the life history of sharks^19,20,23–27^. Even though it is the ^138^Ba isotope that is usually assayed in studies of calcified structures, ^137^Ba was screened in the vertebrae of *S. lewini* in this study for the sake of comparison with results reported in analogous study^19^. As the less abundant isotope (11.2%^40^), ^137^Ba is slightly more challenging to assay reliably than its counterpart, ^138^Ba (71.7%^40^), however this was not considered as a detrimental flaw to the present study because the use of a less abundant isotope would only increase the magnitude of change of the Sr:Ba ratio, though not the general pattern.

**Figure 2:**
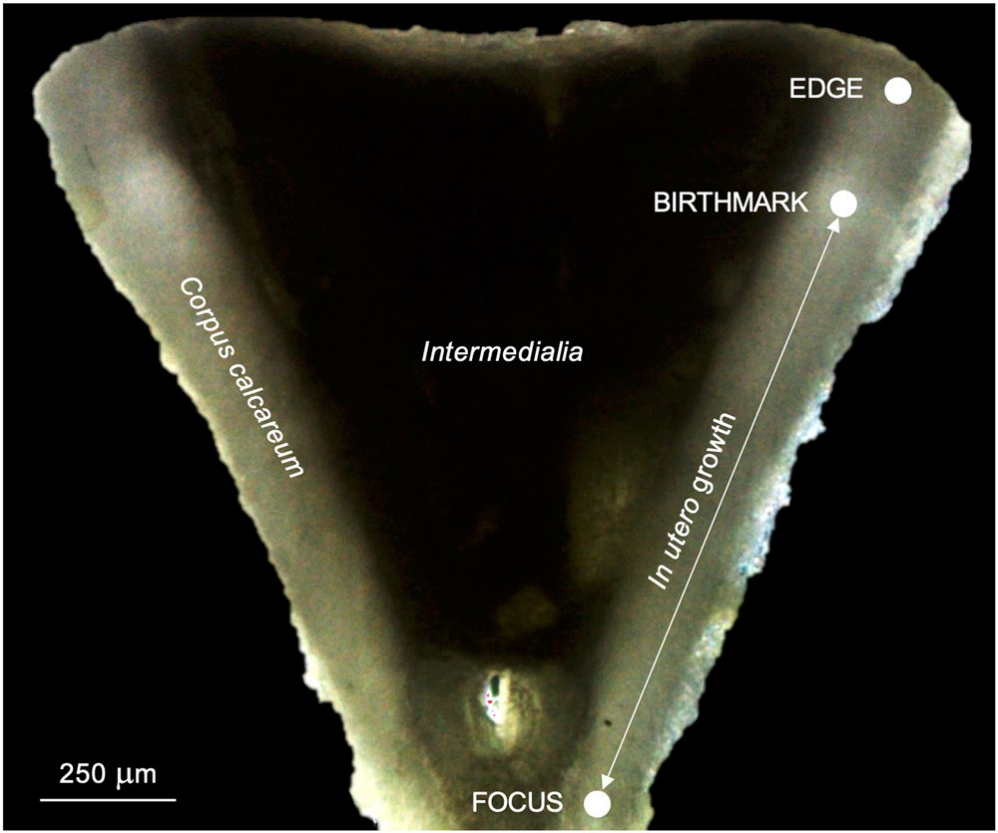
Sagittal section of a vertebra centrum from a young-of-the-year specimen of *Sphyrna lewini*, illustrating the terminology used for LA-ICP-MS analyses.

The consistency in vertebral microchemistry among embryos within each pregnant female’s litter was tested before determining whether the elemental signatures deposited in the vertebrae of embryos reflected maternal vertebral microchemistry (Hypothesis 1; Table 3). Spots targeting the vertebral focus and edge of each embryo were ablated in order to quantify the elemental signatures deposited at the beginning of the gestation and at the time immediately prior to capture, respectively, and test this hypothesis (Fig. 2).

**Table 3:** Summary of the methodology employed to address the different hypotheses of this study.

Hypothesis	Vertebrae	Ablation samples
1. Elemental signatures in vertebrae of embryos from a same litter are similar	EMBRYOS	SPOTS (Focus and Edge)
2. Elemental profiles in vertebrae of embryos from a same litter are similar	EMBRYOS	TRANSECTS (Focus - Edge)
3. Elemental signatures in vertebrae of a mother match with those of her embryos	EMBRYOS vs MOTHERS	SPOTS (Edge)
4. *In utero* elemental profiles in the offspring’s vertebrae reflect migration patterns of pregnant females	YOY	TRANSECTS (Focus – Birthmark)

See Fig. 2 for the target areas of the vertebral sections (*i.e* focus, birthmark or edge) from the embryo, pregnant female and young-of-the-year (‘YOY’) specimens.

The temporal consistency in elemental profiles among embryos within each pregnant female’s litter was also examined to verify that results obtained separately for the focus and the edge in the vertebrae of embryos were consistent for the time period between the beginning of the gestation and the time immediately prior to capture (*i.e. in utero* embryonic development) (Hypothesis 2; Table 3). Transects encompassing the area from the focus to the edge in the vertebrae from each embryo were ablated to characterize the individual elemental profiles and test this hypothesis (Fig. 2).

The hypothesis that vertebral microchemistry of embryos reflected that of pregnant females during the gestation period was tested by comparing the elemental signatures deposited at the vertebral edge of each pregnant female with her respective embryos (Fig. 2), as this region of the vertebrae provided a known spatial and temporal reference (*i.e*. time immediately prior to capture) that made the comparisons possible (Hypothesis 3; Table 3). Spots targeting the vertebral edge of each pregnant female were ablated so the resulting elemental signatures could be compared with the elemental signatures of vertebral edge previously quantified in their embryos.

Ultimately, the potential to infer the gestation-related movements of pregnant females using the *in utero* vertebral microchemistry of their offspring as a proxy was evaluated by measuring changes in ^88^Sr, ^137^Ba and ^208^Pb along transects encompassing complete *in utero* embryonic development from the focus to the birthmark (Fig. 2) of the young-of-the-year vertebrae (Hypothesis 4; Table 3). Strontium (^88^Sr) and barium (^137^Ba) were used as salinity change indicators of the pregnant female environmental histories because: (1) adult females of *S. lewini* primarily inhabit fully marine offshore habitats^8–10^ and (2), nursery areas of the species (*i.e*. estuaries and coastal bays^2,4,14,41–43^) are typically characterized by significant freshwater inputs during the birthing season (*i.e*. May-August^2,3,11^) in the Mexican Pacific^44^, which was expected to influence the nearshore-offshore gradient of salinity and hence the Sr and Ba values^29,45,46^ during this period. The *in utero* variations of lead (^208^Pb) in the vertebrae were assessed as an alternate indicator of contaminated habitat use to aid in interpreting the observations based on ^88^Sr and ^137^Ba.

Spots were ablated with *n* = 3 replicates; circular spot size was 83 μm with a laser repetition rate of 5 Hz and a 60 s duration. Transects were pre-ablated to remove possible external contamination. Pre-ablation scan speed was 108 μm.s^–1^, with a repetition rate of 2 Hz and a 108 μm spot size. For data acquisition, ablation scan speed was 10 μm.s^–1^, with a repetition rate of 10 Hz and an 83 μm spot size. All ablation samples were entirely positioned within and along the *corpus calcareum* of the vertebral sections (Fig. 2).

Elemental data were acquired using an ICP-MS which employed Agilent Technologies ChemStation software operating in time-resolved analysis mode to collect raw data (cps) for the 21 target elements. NIST-612 silicate glass served as external calibration reference material^47^ and was ablated with two replicates before and after every fifth vertebral section was sampled. MACS-3 microanalytical carbonate standard material^48^ was ablated in brackets before and after each master slide to estimate experiment-wide levels of precision. Background data corresponding to gas blanks were collected for 60 s before and after each spot or transect scan was performed. Prior to data acquisition, the ICP-MS instrument was fine-tuned while ablating NIST-612 using 108 µm wide transect scans running at 5 Hz and 86% power in order to maximize element counts and minimize noise. All laboratory facilities and instrumentation used for elemental analyses were located on the campus of the College of Marine Science, University of South Florida, St. Petersburg, FL, USA.

### Data analyses

#### Elemental signatures in vertebrae: embryos versus pregnant females

Raw spot data (cps) were visually assessed within the software, and those portions of the signals displaying peaks likely associated with surface contaminants or other forms of instabilities were excluded from further processing. The following operations were then applied to the data associated with each spot sample: (1) background levels were removed by subtraction; (2) mass-specific spikes detected by the Grubbs test (α = 0.05) were replaced with mean values; and (3) mass-specific drift in the sensitivity of the ICP-MS detector was corrected via linear interpolation. Raw spot data (cps) were then converted to single, mean (*i.e*. averaged across replicates) elemental concentration values (ppm) using NIST-612 data for external calibration and standardized to the ^43^Ca data obtained simultaneously in the structure by deriving element:Ca ratios (µmole⋅mole^−1^) to adjust for variability in instrument sensitivity and the amount of ablated material. Limits of detection (LOD) were estimated for samples based on 3⋅SD of the ion count rates (cps) of the corresponding gas blanks and converted to concentrations (ppm). Elements with ≥10% of the measures of concentration below LOD were omitted from subsequent analyses. Outliers among replicate spot scans were identified using a multivariate measure of outlyingness^49^ based on elemental concentrations (ppm). Replicates with outlyingness values >10 were excluded before reducing the data to mean ppm concentration value for each sample. Raw transect data (cps) were made compatible for direct comparisons by interpolating cell array of each vertebral transect so they all had the same number of data points as the longest one and generating mean integrated transect data of the retained elements (*i.e*. consistently recorded above LOD).

The null hypotheses that (1) no difference existed in the elemental signatures deposited at the vertebral focus and edge of embryos within each pregnant female’s litter, (2) no difference existed among the elemental profiles of embryos within each litter, and that (3) no difference in the elemental signatures of edge existed between a pregnant female and her embryos were tested using permutational analysis of variance (PERMANOVA^50^). Canonical analyses of principal coordinates (CAP^51^) were subsequently employed to visualize the within-group similarities detected using PERMANOVA and test the ability of the models to accurately distinguish among pregnant females and their corresponding embryos based on vertebral microchemistry. Leave-one-out cross-validation (LOO-CV) was used to assess the overall classification accuracy of each CAP model and build a confusion matrix summarizing the occurrence of group-specific misclassifications. Proportional chance criterion (PCC) was used to test the significance of the observed overall classification success rates of the CAP models compared with that expected by chance^52^.

Spot (*i.e*. focus and edge) and transect data were analyzed as multi-elemental signatures of the mean (*i.e*. averaged across replicates) element:Ca ratios and mean integrated transect data of the retained elements, respectively. A Euclidean distance-based dissimilarity matrix constructed from the mean element:Ca ratios or elemental profiles served as multiple, quantitative explanatory variables in PERMANOVA and CAP design, with individual pregnant females and/or litters serving as the categorical response variables.

#### Inferring the gestation-related movements of pregnant females

Transect data for ^88^Sr,^137^Ba and ^208^Pb (used as Sr:Ba and Pb:Ca raw cps ratios) were plotted versus vertebral transect distance from the focus (μm) of each young-of-the-year to evaluate the viability to employ the *in utero* elemental profiles of *S. lewini* to infer the gestation-related movements of pregnant females. Data were used as raw cps for the sake of comparison with analogous study involving *S. lewini* in the Mexican Pacific^7^. An 11-point running average window size was used to filter/smooth the data, reduce the noise, and aid in identifying the underlying pattern of the Sr:Ba and Pb:Ca profiles^53^. The same procedure was subsequently applied to the embryo transect data to compare the patterns observed in their elemental profiles with those generated from the young-of-the-year while *in utero*. Evidences of pregnant females possibly entering estuarine or nearshore habitats for parturition were expected to be shown by a sharp decline of the Sr:Ba ratio (between 150 and 400^7^) in the *in utero* profiles prior to birth combined with an increase of the Pb:Ca ratio at the same time^7^. Although previous study recently confirmed that Sr:Ca and Ba:Ca were inversely related relative to each other in the vertebrae of *S. lewini* relative to the nearshore-offshore environmental gradient in the region of study^7^, this assumption was also verified in the present study before inferences on the movements of pregnant females could be made based on their offsprings’ *in utero* Sr:Ba profiles.

When a marked shift was observed in the *in utero* profiles of a sample, the total length of the individual at that time *t* of the embryonic development was estimated using the back-calculation Fraser–Lee method^54^ (1):

1 $${L}_{t}=[({R}_{t}){({R}_{V})}^{-1}]({L}_{C}-{\rm{a}})+{\rm{a}}$$

where *L*_t_ is the back-calculated total length corresponding to age *t* (*i.e*. time of the pregnant female habitat shift), *R*_t_ corresponds to the vertebral transect distance from the focus to the elemental shift identified *in utero* at age *t*, *R*_*V*_ is the vertebrae centrum radius, *L*_C_ the total length at the time of capture of the individual (*i.e*. young-of-the-year or embryo) and *a* is intercept of an established linear relationship between *R*_V_ and *L*_C_^54^, which is *L*_C_ = 17.349 *R*_*V*_ + 14.516 for juvenile specimens of *S. lewini* in the Mexican Pacific^55^.

The Fraser–Lee equation was selected as a back-calculation method in order to correct for bias induced when the *R*_V_-*L*_C_ linear regression does not pass through the origin (such as is the case for *S. lewini*) by using the intercept *a* estimated from the *R*_V_-*L*_C_ relationship as a correcting factor in Eq. (1) ^56^. Estimating the *in utero* back-calculated length at the time of the pregnant female habitat shift was considered necessary in this study to emphasize the importance of protecting these individuals when entering nearshore habitats a few cm before the embryos have reached a full-term total length. Although back-calculation may not adequately describe faster embryonic growth during the early stages of the gestation^57^, this was not considered as an issue in the present study because the *in utero* total lengths of the individuals at the time of the pregnant female habitat shift were estimated for the period corresponding to the late stages of the gestation and the parameters used in Eq. (1) were derived from juveniles, which allowed to avoid bias induced by mature individuals exhibiting different growth rates^58^.

All elemental data processing and multivariate statistical analyses were performed using the free download Fathom Toolbox for Matlab^TM^^59^. For more details on the data processing and multivariate statistical analyses, please refer to the readme files and corresponding Matlab codes available on GitHub.

### Compliance with ethical standards

All sample collection was carried out in accordance with relevant national guidelines and regulations for the care and use of animals. The samples used for this study were obtained from dead specimens of scalloped hammerhead sharks destined for human consumption, which were legally caught by fishers in possession of the proper fishing permits issued by the National Commission for Aquaculture and Fisheries from the Mexican Ministry of Agriculture and Rural Development (CONAPESCA: https://www.gob.mx/conapesca), and in agreement with the Mexican Official Regulation NOM-029-PESC-2006 regarding the sustainable exploitation of sharks and rays (NORMA Oficial Mexicana NOM-029-PESC-2006, Pesca responsable de tiburones y rayas. Especificaciones para su aprovechamiento, https://www.gob.mx/cms/uploads/attachment/file/135366/49.-_NORMA_OFICIAL_MEXICANA_NOM-029-PESC-2006.pdf).

## Results

### Elemental signatures in vertebrae: embryos versus pregnant females

The vertebrae from 4 pregnant females of *S. lewini* captured off Puerto Madero and 3 to 4 embryos from their respective litters were available for this study (Hypotheses 1–3; Table 3). Pregnant females ranged from 240 to 291 cm of *L*_T_ (mean ± SD = 265 ± 23 cm *L*_T_; Table 1). Embryos ranged from 41 to 49 cm of *L*_T_ (*n* = 14; 44.8 ± 2.8 cm *L*_T_; Table 1). Based on the reported length-at-birth of *S. lewini* in the Mexican Pacific (*i.e*. 41–53 cm *L*_T_^2–4,11^), these embryos were about to be born.

The mean percentage of the elemental concentration data below the limits of detection (LOD) was ≤10% for all elements (*i.e*. ^7^Li, ^24^Mg, ^55^Mn, ^57^Fe, ^59^Co, ^85^Rb, ^88^Sr, ^89^Y, ^114^Cd, ^118^Sn, ^137^Ba, ^208^Pb), except for ^45^Sc (42.9%), ^51^V (57.1%), ^53^Cr (64.3%), ^72^Ge (71.4%), ^89^Y (64.3%), ^114^Cd (57.1%), ^197^Au (71.4%), ^232^Th (63.3%) and ^238^U (78.6%) which were not regularly detected in the vertebrae of embryos and pregnant females. For more details on the mean (±standard deviation) element:Ca ratios (µmole⋅mole^−1^) quantified at the vertebral focus and edge of embryos and pregnant females, please refer to Table S1 from the Supplementary Information.

**Figure 3:**
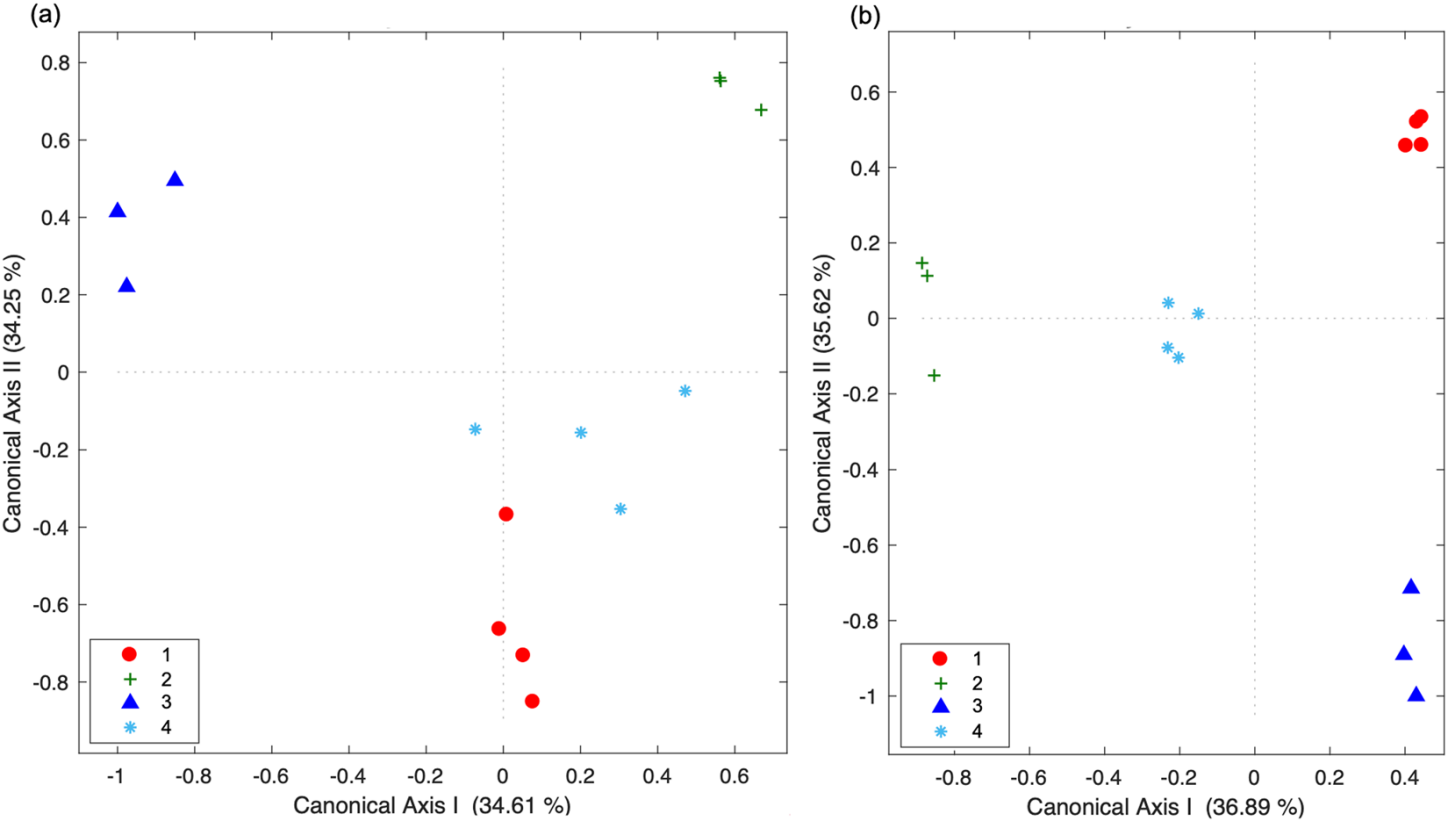
Canonical analyses of principal coordinates ordination diagrams illustrating the spatial variation of **(A)** multi-elemental signatures deposited at the vertebral focus (CAP_FOCUS_) and **(B)** multi-elemental profiles (CAP_TRANSECT_) quantified in the embryos of *Sphyrna lewini* (*n* = 14). Numbers refer to the female’s litter codes (see Table 1).

The multi-elemental signatures deposited at the vertebral focus of embryos were consistent within each pregnant female’s litter (PERMANOVA: 0.68 < *P* < 1; Hypothesis 1), 85.7% of the embryos were correctly assigned within each corresponding litter by the CAP_FOCUS_ model classifier (CAP_FOCUS_: *m* = 8, *G*_prop_ = 100%, *Trc*_stat_ = 2.66, *P* = 0.001; Fig. 3A) and this was significantly better than the 25.5% accuracy rate expected by chance (PCC: *P* = 0.001).

The multi-elemental profiles were also consistent among embryos within each pregnant female’s litter (PERMANOVA: 0.36 < *P* < 1, Hypothesis 2); 60% of the embryos were correctly assigned within each corresponding litter by the CAP_TRANSECT_ model classifier (CAP_TRANSECT_: *m* = 11, *G*_prop_ = 86.9%, *Trc*_stat_ = 2.7, *P* = 0.04; Fig. 3B) and this was significantly better than the 40% accuracy rate expected by chance (PCC: *P* = 0.04).

**Figure 4:**
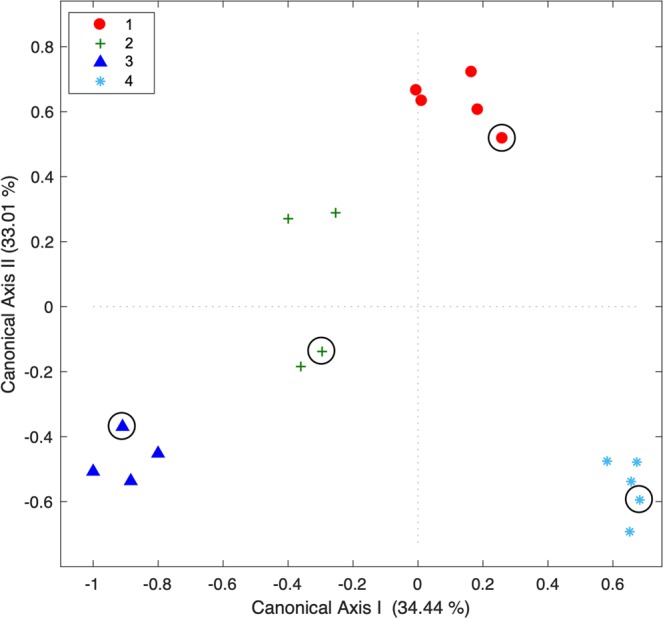
Canonical analysis of principal coordinates (CAP_EDGE_) ordination diagram illustrating the spatial variation of elemental signatures deposited at the vertebral edge of the pregnant females of *Sphyrna lewini* (*n* = 4) and their embryos (*n* = 14). Numbers refer to the female’s litter codes (see Table 1). Symbols with black circles correspond to the litter’s female.

The multi-elemental signatures deposited at the vertebral edge were consistent between each pregnant female and her embryos (PERMANOVA: *P* = 1; Hypothesis 3); 88.9% of the samples were correctly assigned within each corresponding group (combining embryos and pregnant females) by the CAP_EDGE_ model classifier (CAP_EDGE_: *m* = 12, *G*_prop_ = 100%, *Trc*_stat_ = 2.85, *P* = 0.001; Fig. 4), which was significantly better than the 26.4% accuracy rate expected by chance (PCC: *P* = 0.001). For details on the elements driving most of the differences among elemental signatures deposited at the vertebral focus and edge of embryos and pregnant females, please refer to Figs. S1 and S2 from the Supplementary Information.

### Inferring the gestation-related movements of pregnant females

The vertebrae from 15 young-of-the-year specimens of *S. lewini* ranging from 49.2 to 59.6 cm of *L*_T_ (mean ± SD = 55.7 ± 4.1 cm *L*_T_; Table 2) were available for this study. Elemental profiles quantified in their vertebrae were compared with those previously quantified in the vertebrae of the 14 near-term embryos (Table 1).

**Figure 5:**
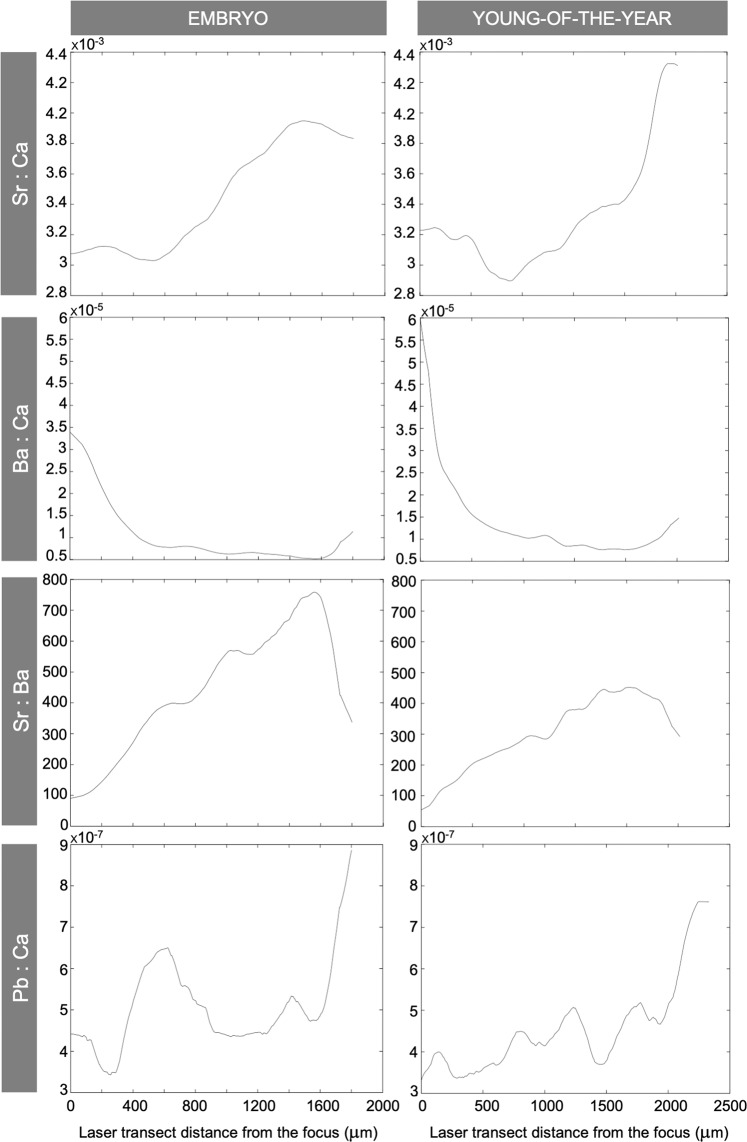
Individual *in utero* profiles of the Sr:Ca, Ba:Ca, Sr:Ba and Pb:Ca ratios quantified in the vertebrae of an embryo (left) and a young-of-the-year (right) specimen of *Sphyrna lewini*.

All individuals (*i.e*. embryos and young-of-the-year) exhibited the same pattern of variation in their *in utero* Sr:Ca and Ba:Ca profiles. The Sr:Ca ratio progressively increased throughout gestation and eventually slightly declined prior to parturition whereas the Ba:Ca ratio drastically declined at the beginning of the gestation (*i.e*. focus), remained particularly low and constant during its intermediate stage and then increased prior to birth (*i.e*. vertebral edge or birthmark; see example in Fig. 5). Although Sr:Ca and Ba:Ca were effectively inversely related relative to each other, Sr:Ba alone provided a better tool for examining the individual environmental histories rather than the joint comparison of the Sr:Ca and Ba:Ca ratios because it allowed to combine and highlight the differences observed for both (Fig. 5). Regarding variations of Pb along the *in utero* profiles, all samples exhibited a sharp increase of the Pb:Ca ratio prior to birth when compared to earlier in the gestation (Fig. 5). Accordingly, Sr:Ba was used as primary environmental indicator combined with Pb:Ca in order to support the observations based on Sr and Ba.

**Figure 6:**
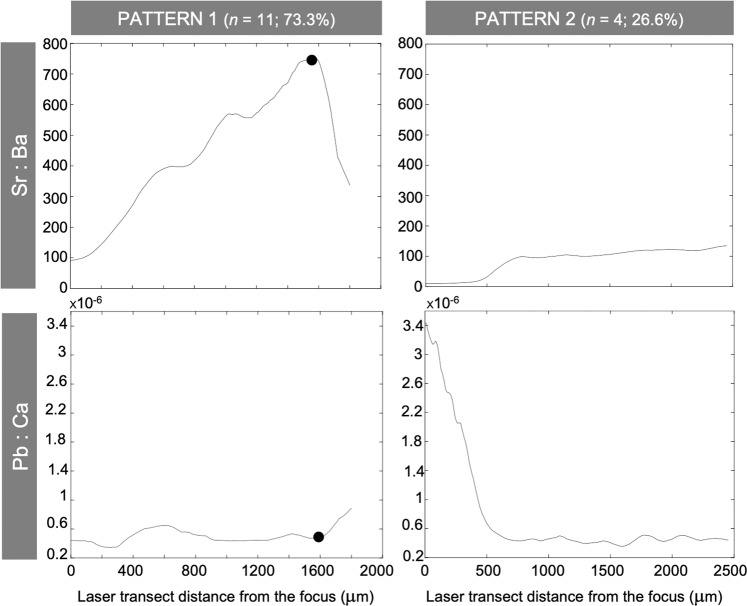
Examples of the *in utero* Sr:Ba and Pb:Ca ratio profiles quantified in the vertebrae from two young-of-the-year specimens of *Sphyrna lewini*, illustrating the two patterns identified (*i.e*. ‘Pattern 1’ and ‘Pattern 2’). Dots indicate possible habitat shift of pregnant females towards nearshore habitats.

Embryos and young-of-the-year of *S. lewini* displayed large variations in Sr:Ba while *in utero*, with overall values that could vary from <100 to 1200 within individuals, suggesting that pregnant females remained in environments of contrasting salinities throughout gestation (Fig. 5). The Sr:Ba ratio also differed widely among individuals, with the maximum individual values ranging between 300 and 1200 and the samples from Salina Cruz displaying the highest (1050–1200). Overall, all embryos and young-of-the-year exhibited relatively low Sr:Ba values at the vertebral focus (0–200) while the Sr:Ba values quantified at the vertebral birthmark of the young-of-the-year were more variable (100–600) among individuals (Figs. 5 and 6).

A single pattern was found in the *in utero* Sr:Ba profiles of embryos (subsequently referred to as ‘Pattern 1’); Sr:Ba gradually increased until the highest individual value was reached (between 600 and 1300) and then rapidly declined to values between 300 and <100 shortly prior to the female capture, which corresponded to a sharp increase of the Pb:Ca ratio at the same time (Fig. 5). Back-calculated length of embryos at the time of the decline in Sr:Ba ranged between 37.6 cm and 45.9 cm of *L*_T_ (41.1 ± 2.1 cm *L*_T_), which corresponded to a difference of size of approximately 4.2 ± 1.9 cm of *L*_T_ with that of the embryos at the pregnant female capture.

The same pattern of variation was observed in the *in utero* Sr:Ba profiles of most young-of-the-year (*n* = 11; 73.3%) (*i.e*. ‘Pattern 1’; Fig. 6); Sr:Ba gradually increased until the highest individual value was reached (600–1200), and then rapidly declined to values between 350 and <100 shortly prior to birth, which also corresponded to an increase of the Pb:Ca ratio at the same time (Fig. 6). The *in utero* back-calculated length at the time of the Sr:Ba decline ranged between 36.3 and 52.3 cm of *L*_T_ (43.9 ± 5.4 cm *L*_T_) for these samples, which was not significantly different from that estimated for the embryos (*i.e*. 41.1 ± 2.1 cm; ANOVA: *F* = 2.57, *P* > 0.05). The remaining young-of-the-year specimens (*n* = 4; 26.6%) exhibited low but relatively constant values of the Sr:Ba ratio (0–200) while *in utero* (subsequently referred to as ‘Pattern 2’; Fig. 6), suggesting that these specimens’ mothers possibly remained nearshore throughout gestation. These specimens were also found to exhibit significantly higher values of the Pb:Ca ratio at the beginning of the gestation when compared to later in the embryonic development (*i.e*. focus; 1.25 × 10^–6^ ± 1.03 × 10^−6^ versus 0.46 × 10^−6^ ± 0.77 × 10^−6^ in specimens exhibiting ‘Pattern 1’; Mann–Whitney test: *P* < 0.001), after which the ratio remained relatively constant (Fig. 6).

## Discussion

### Elemental signatures in vertebrae: embryos versus pregnant females

Vertebral microchemistry of pregnant females of *S. lewini* and some of their near-term embryos was analyzed in this study to address important questions concerning the gestation-related migratory patterns of the species in the Mexican Pacific. Previous studies recently demonstrated that elemental signatures in the vertebrae of *S. lewini* can serve as broad-scale effective spatial markers in the Mexican Pacific whether individuals have occupied coastal or oceanic environments and irrespective of the ontogenetic stage^7,23^, and the results of this study indicated that elemental signatures of the species can also serve as maternal tags, when these are quantified in the area of the vertebrae that was deposited during the *in utero* embryonic development. Although only few samples could be collected, vertebral microchemistry of *S. lewini* allowed to successfully distinguish among embryos from the distinct litters (60–88.9%), whether based on discrete elemental signatures deposited at the vertebral focus and edge (Hypothesis 1; Table 3) or elemental profiles that encompassed complete *in utero* development (Hypothesis 2; Table 3), demonstrating the temporal consistency in the elemental deposition among embryos of the species within a same litter.

Vertebral microchemistry of embryos was also found to reflect that of pregnant females during the gestation (Hypothesis 3; Table 3), at least at the edge (*i.e*. region of the vertebrae deposited immediately prior to capture), as this region of the vertebrae provided the only known spatial and temporal reference from which the comparisons could be made. Considering to compare elemental signatures deposited at another time such as the beginning of the gestation (*i.e*. focus) was not possible because there is no temporal reference in the vertebrae of pregnant females allowing to calibrate the period of the gestation in terms of months (*i.e*. 10–11 months^11,12^). Nevertheless, the fact that elemental signatures were temporally consistent among embryos within each litter and that vertebral microchemistry allowed to successfully classified (88.9%) pregnant females with their respective embryos based on their unique elemental signatures of vertebral edge indicated that *in utero* vertebral microchemistry of *S. lewini* can serve as maternal tag from a few weeks after fertilization, when the formation of a placenta allows the embryos to be directly nourished from the mother’s blood stream, to the end of the gestation.

It was recently stated that pregnant female’s transfer of substantial amounts of organic contaminants (*i.e*. polychlorinated biphenyls and chlorinated pesticides) to their embryos through the process of fetal nutrition^60^ might potentially alter the maternal site-specific markers deposited in the vertebrae of embryos while *in utero*^23^. Although it is unknown whether maternal contaminant offloading can effectively alter the vertebral microchemistry of embryos of *S. lewini*, the lipophilic nature of organochlorines does not support this assumption as such contaminants primarily accumulate within the hepatic tissue^61,62^. Furthermore, the fact that in this study vertebral microchemistry of pregnant females was not significantly different from that of their embryos indicated that elemental signatures in vertebrae of embryos are likely not altered through the mechanisms of contaminant transfer, thus confirming that *in utero* elemental signatures of *S. lewini* can serve as intrinsic markers of the environmental histories of pregnant females during the gestation.

### Inferring the gestation-related movements of pregnant females

Conventional tagging studies indicated that adult females of *S. lewini* typically aggregate offshore and later return to coastal habitats for parturition^8–10^. Although the patterns of migration and habitat use of pregnant individuals have remained unknown despite extensive study, yet it could be assumed that the female reliance on coastal habitats for parturition combined with an annual reproductive cycle^11,12^ had restricted their dispersal to highly oceanic habitats during the gestation. The novel approach developed in this study allowed to elucidate some of these aspects through the analysis of the female offspring’s *in utero* vertebral microchemistry, using Sr:Ba and Pb:Ca as indicators of environmental histories (Hypothesis 4; Table 3). Even though the lack of water chemistry data and telemetric data against which to compare the results of this study limits the power of such inferences, analyses of the *in utero* Sr:Ba and Pb:Ca profiles allowed to detect two apparently distinct migratory patterns of the pregnant females of *S. lewini* in terms of the movements between nearshore and more oceanic environments.

More specifically, the results indicated that most pregnant females (73.3%) apparently progressively migrated offshore from the initial stages of the gestation (*i.e*. a few weeks after fertilization) before quickly heading back to the coastal nurseries prior to parturition (‘Pattern 1’), when embryos had reached a size between approximately 41.1 and 43.9 cm of *L*_T_. Similarities in such habitat shift among pregnant females at this time of the gestation were shown by marked decline of the Sr:Ba ratio to values between 300 and <100 prior to birth in the *in utero* Sr:Ba profiles of young-of-the-year and embryos, in accordance with previously observed changes in the vertebral microchemistry of adult specimens of *S. lewini* when entering nearshore habitats (150–400)^7^, assuming that variations in Sr and Ba effectively reflect the gradient of salinity changes of the environment^17,29^.

These observations were further informed by the analyses of the *in utero* Pb:Ca profiles that showed a sharp increase of the ratio prior to birth in both embryos and young-of-the-year, possibly indicative of pregnant females entering nearshore contaminated habitats for parturition, even though the assumption that incorporation of Pb in the vertebrae effectively reflects the exposure of a shark to contamination derived from anthropogenic sources has not been explicitly tested. Nevertheless, the fact that (1) embryos of pregnant females of *S. lewini* captured nearshore were found in this study to exhibit particularly higher Pb:Ca values at the vertebral edge when compared to earlier in the gestation, and that (2) ^208^Pb in the vertebrae of *S. lewini* was found in previous study to be characteristic of the specimens captured in nearshore areas^7^ strongly influenced by anthropogenic sources of trace metal and pollutant inputs^63–65^ (when compared to individuals captured offshore^7^) indicated that Pb could be used as a complementary indicator of environmental history of *S. lewini* to support the conclusions based on Sr and Ba.

Taking this into account, the results of this study also suggested that some pregnant females (23.3%) apparently remained nearshore during complete gestation, as shown by low but constant values of the Sr:Ba ratio (<200) along their offspring’s *in utero* profiles, associated with relatively high but constant values of the Pb:Ca ratio (‘Pattern 2’). This possible behavior has not been documented before for *S. lewini* and the reasons why some females presumably remained nearshore during the gestation are unknown, especially considering that adult stages of *S. lewini* are thought to migrate offshore as a strategy to increase their foraging success by feeding on energy-rich pelagic preys^5^. Nevertheless, the fact that only a small proportion (26.6%) of the individuals were found to exhibit this pattern precluded robust conclusion regarding this point, especially since sample size available for this study was in fact relatively low.

On the other hand, it might be stated that the among-individual differences observed in the Sr:Ba patterns and shifts might be driven by other factors than salinity such as variations in temperature, dietary preferences or individual-specific physiology (as shown for some teleosts^66,67^), and hence be not fully reflecting the movements of females across the nearshore-offshore gradient of salinity change. However, experimental evidences showed that: (1) Sr and Ba are not physiologically regulated and their incorporation in the vertebrae being primarily derived from branchial uptake, representative of the environmental concentrations, but not affected by somatic growth nor vertebral precipitation rate^16,18^ and (2), that temperature positively affected the incorporation of both Sr and Ba in the vertebrae of sharks^18^, and hence would not affect the overall Sr:Ba ratio variations if females were to move into warmer (or cooler) waters during the gestation, regardless of the nearshore-offshore gradient of salinity. Caution should be however taken regarding the latter because conflicting evidences on the effect of temperature were found with round stingray *Urobatis halleri*, as temperature negatively affected the incorporation of Ba, though not that of Sr, in its vertebrae^16^. Nevertheless, analyses of the Pb:Ca ratio were used in this study as a complementary indicator of environmental history to further evidence the movements of pregnant females into the nearshore habitats, and the results were consistent with the observations based on Sr:Ba.

It was recently suggested that in the Mexican Pacific females of *S. lewini* may probably give birth in variable environments and/or that the use of coastal nurseries may be less defined for this species than previously assumed^7^, and the fact that in this study Sr:Ba values quantified at the young-of-the-year vertebral birthmark was found to differ widely among individuals (<100–600) also supports this assumption. Behavioral plasticity towards nursery habitat selection for parturition might effectively be a strategy of the females to enhance their offspring survival by differing its vulnerability to predators and a potential lack of foraging success among regions^7^.

It is also important to note that even if the results of this study suggested that females remained nearshore at the beginning of the gestation because the Sr:Ba values quantified at the vertebral focus were found to be particularly low (50–200) in all individuals (while Pb:Ca was comparatively higher), conclusions regarding this point remain tentative because elemental data for this area of the vertebrae might primarily reflect vitellogenesis (*i.e*. yolk deposition) rather than the actual initial stage of the gestation, as placentation generally occurs within a few weeks after fertilization^34^. Yolk-based nourishing of embryos during the first weeks of gestation may possibly lead to a lag in the time required for the embryo vertebral microchemistry to reflect variations in the chemistry of the surrounding water to which the pregnant female was exposed during these weeks. Accordingly, the *in utero* elemental profiles of *S. lewini* may be informative on the migration patterns of pregnant females only once embryos were directly fed through the mother’s blood stream, a few weeks after fertilization^34^.

Nevertheless, yet it appeared that pregnant females of *S. lewini* likely exhibited two distinct migratory patterns in the Mexican Pacific, as they either (1) progressively migrated offshore before quickly returning to coastal nurseries before term, or (2) possibly remained nearshore during complete embryonic development, supporting the assumption that overall females might exhibit relatively short dispersal to the oceanic habitats during the gestation. Alternating between two migratory patterns during the gestation may be a strategy of females to reduce their vulnerability to stressors or unfavorable environmental conditions among regions^68–70^ and optimize chances to complete a full-term gestation.

Considering the life history and global endangered status of *S. lewini*^71^, current management measures in the Mexican Pacific such as the regulation norm of the commercial shark fishery (NOM-029-PESC-2006^72^) and its seasonal prohibition between May and July (NOM-009-SAG/PESC-2015^73^) may be insufficient for the sustainable management of the population. Pregnant females may be particularly susceptible to fisheries when remaining nearshore or entering coastal nurseries slightly before term, therefore limiting their possibilities to complete gestation to the term^2^ and reducing the reproductive potential of the population as it is briefly centralized in coastal areas^23^. Additional information on the movements of reproductive females provided by fine-scale telemetric studies is required to determine how such strategies of migration could potentially impact the reproductive potential of the population.

## Supplementary information

Supplementary Information.

## Data Availability

The Matlab codes used to undertake the analyses performed in this study and prepare the figures and tables presented is freely available on GitHub (https://github.com/clairecoiraton/HammerheadInUteroMicrochemistry_MatlabCode.git).
