# Supplementary material for: In utero elemental tags in vertebrae of the scalloped hammerhead shark Sphyrna lewini reveal migration patterns of pregnant females
Source: Sci Rep. 2020 Feb 4;10:1799. doi: 10.1038/s41598-020-58735-8 (PMC7000759; doi:10.1038/s41598-020-58735-8)

Supplementary Information

Coiraton C., Amezcua, F. *In utero* elemental tags in vertebrae of the scalloped hammerhead shark *Sphyrna lewini* reveal migration patterns of pregnant females.

Table S1. Mean (± standard deviation) element:Ca ratios (μmole·mole^{-1}) quantified at the vertebral focus and edge of embryos and pregnant females of *Sphyrna lewini* captured in April 2016 off Puerto Madero in the Mexican Pacific.

ELEMENT	FOCUS		EDGE (EMBRYOS)		EDGE (MOTHERS)	
	MEAN	± SD	MEAN	± SD	MEAN	± SD
Li7	1.05	0.18	1.35	0.34	1.32	0.17
Na23	6241.42	1033.46	7963.09	2591.18	7327.39	2032.91
Mg24	4746.61	480.22	6202.11	1065.93	6877.59	328.29
Mn55	30.56	7.38	19.35	6.94	20.87	3.67
Fe57	185.14	10.56	164.74	30.18	180.28	5.04
Co59	0.25	0.07	0.20	0.15	0.23	0.04
Cu63	48.62	61.52	44.63	72.85	18.91	16.27
Rb85	1.26	0.74	1.04	1.25	1.74	1.39
Sr88	971.25	71.42	1463.65	318.60	1285.39	80.50
Sn118	0.94	1.60	0.47	0.65	0.28	0.07
Ba137	14.44	5.25	1.97	1.53	1.60	0.97
Pb208	0.34	0.30	0.24	0.05	0.28	0.06

Figure S1. Canonical analysis of principal coordinates (CAP$_{FOCUS}$) ordination diagram and vector plot illustrating the spatial variation of multi-elemental signatures deposited at the vertebral focus quantified in the embryos of *Sphyrna lewini* (*n* = 14). Numbers refer to the female's litter code (see Table 1).

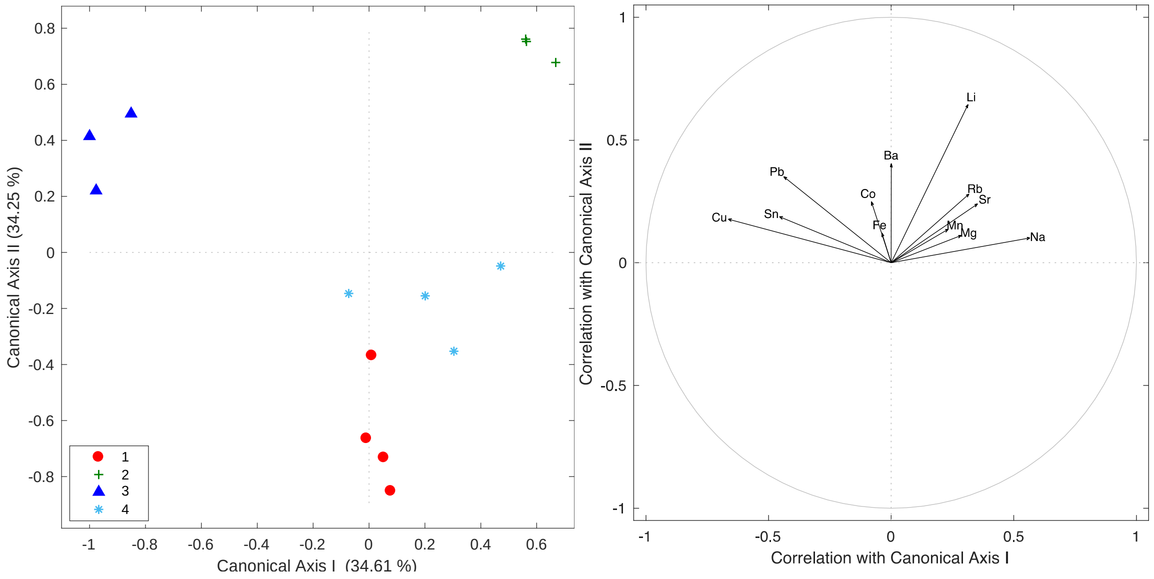

Figure S2. Canonical analysis of principal coordinates (CAP_{EDGE}) ordination diagram and vector plot illustrating the spatial variation of elemental signatures deposited at the vertebral edge of the pregnant females of *Sphyrna lewini* (*n* = 4) and their embryos (*n* = 14). Numbers refer to the female's litter codes (see Table 1). Symbols with black circles correspond to the litter's female.

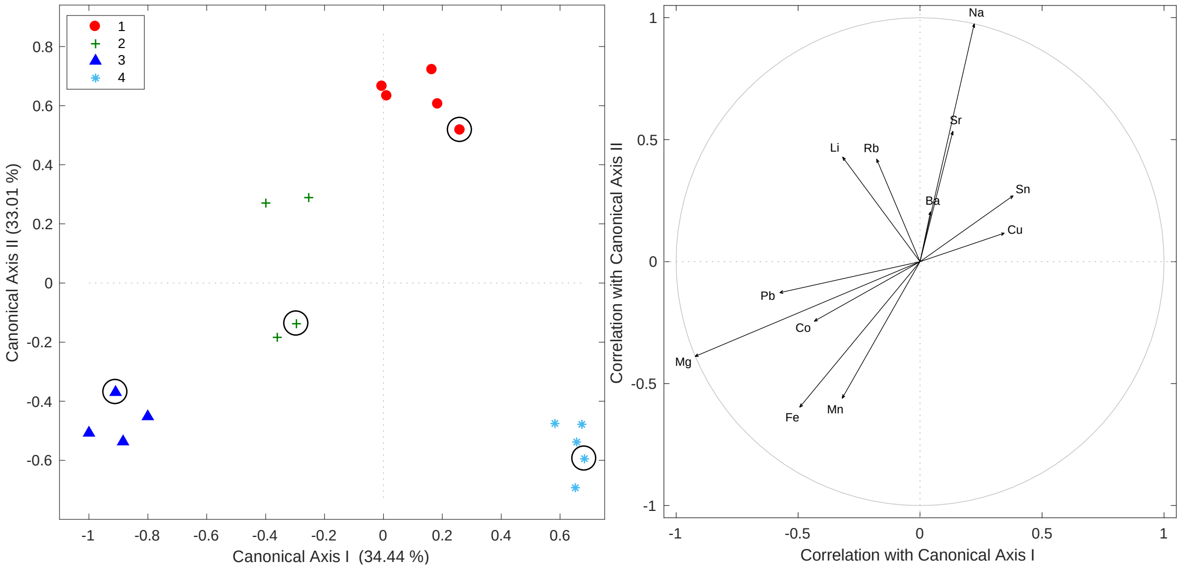

Supplement: Supplementary file 1 — Supplementary Information. [file 41598_2020_58735_MOESM1_ESM.pdf]
